# miR-125b-5p inhibits cell proliferation, migration, and invasion in hepatocellular carcinoma via targeting *TXNRD1*

**DOI:** 10.1186/s12935-019-0919-6

**Published:** 2019-07-30

**Authors:** Shengni Hua, Yingyao Quan, Meixiao Zhan, Huaxin Liao, Yong Li, Ligong Lu

**Affiliations:** 1 Zhuhai Interventional Medical Center, Zhuhai Precision Medical Center, Zhuhai People’s Hospital, Zhuhai Hospital Affiliated with Jinan University, Zhuhai, 519000 China; 2 Biomedicine Institute, College of Life Science, Jinan University, Guangzhou, 510632 China

**Keywords:** miR-125b-5p, Hepatocellular carcinoma, Proliferation, TXNRD1

## Abstract

**Background:**

*Thioredoxin reductase 1* (*TXNRD1*) is an antioxidant enzyme reportedly overexpressed in hepatocellular carcinoma (HCC); however, the detailed function and mechanisms of TXNRD1 in HCC remain obscure. In this study, we investigated the miR-125b-5p-specific regulation of *TXNRD1* levels and its effect on HCC cells.

**Methods:**

We detected miR-125b-5p levels in human HCC tissue samples through quantitative reverse transcription polymerase chain reaction (qRT-PCR), and in vitro experiments were employed to investigate the effect of miR-125b-5p on HCC cell proliferation, migration, and invasion. Additionally, we examined miR-125b-5p-mediated changes in TXNRD1 levels by qRT-PCR and western blotting, and a dual luciferase-reporter assay was conducted to confirm direct targeting of the 3′ untranslated region of TXNRD1 mRNA by miR-125b-5p.

**Results:**

miR-125b-5p expression was reduced in HCC tissues relative to that in matched para-carcinoma tissues; this finding was verified in HCC cohorts from the Gene Expression Omnibus and The Cancer Genome Atlas. Additionally, low miR-125b-5p expression was associated with poor prognosis in HCC patients, and gene-set enrichment analysis indicated that miR-125b-5p levels were associated with HCC proliferation and metastasis. As predicted, overexpressing miR-125b-5p restrained the proliferation, migration, and invasion of Huh7 and SK-Hep-1 cells and forced expression of the miR-125b-5p-downregulated TXNRD1 mRNA and protein levels in HCC cells. Moreover, dual luciferase-reporter assays revealed that miR-125b-5p targets *TXNRD1* to directly regulate its expression, whereas *TXNRD1* overexpression abolishes the inhibitory effect of miR-125b-5p on HCC cell proliferation, migration, and invasion.

**Conclusions:**

These results demonstrated miR-125b-5p as a tumor suppressor in HCC through its inhibition of *TXNRD1*, thereby suggesting it as a potential target for the clinical treatment of HCC.

**Electronic supplementary material:**

The online version of this article (10.1186/s12935-019-0919-6) contains supplementary material, which is available to authorized users.

## Background

Liver cancer is reportedly the 6th most common cancer and the 4th most lethal cause of cancer-related death worldwide, with hepatocellular carcinoma (HCC) accounting for ~ 80% of these cases [1]. Due to various pathogeneses involved in HCC tumor progression, the heterogeneity of HCC cells, and the late stage during which HCC is usually identified, the prognosis of HCC patients is poor and, despite the recent discovery of subclinical molecular targets, the overall survival rate of HCC remains low [2]. Therefore, it is necessary to continue investigating the molecular mechanisms associated with HCC progression in order to identify additional prognostic indicators.

MicroRNAs (miRNAs) are small, noncoding RNAs that participate in the posttranscriptional regulation of gene expression [3], and accumulating data indicate that miRNAs play an important role in modulating multiple biological behaviors associated with cancer [4]. Moreover, miRNA levels are correlated with neoplasm staging, tumor relapse, drug tolerance, and prognosis [5–7]. However, the mechanisms underlying the miRNA-specific regulation of tumor progression have not been completely investigated.

Aerobic organisms exhibit sophisticated antioxidation mechanisms in order to maintain a reducing environment in cells. Thioredoxin-1 (TRX1) is a key contributor to intracellular redox homeostasis and promotes energy and carbohydrate metabolism [8]. Mechanistically, TRX1 responds to reactive oxygen species produced by cellular respiration and metabolism and facilitates the reduction of other proteins at the cost of its own oxidation [9]. Thioredoxin reductase-1 (TXNRD1) is uniquely capable of utilizing electrons from NADPH to recover the reduced state of TRX1. Interestingly, TXNRD1 is upregulated in many human malignancies and promotes cancer progression [10], and attenuation of TXNRD1 levels effectively suppresses the growth of tumor cells [11].

In this study, we investigated the regulatory relationship between miR-125b-5p and *TXNRD1* in HCC cells. Furthermore, we examined the effect of miR-125b-5p on the biological function of HCC cell lines.

## Methods

### Clinical samples

A total of 13 pairs of fresh HCC and matched adjacent tissues were acquired from patients undergoing their first hepatectomy and diagnosed with HCC between May 2016 and June 2018 at the Zhuhai People’s Hospital, Jinan University (Zhuhai, China). None of the patients had received radiotherapy or chemotherapy prior to the hepatectomy. Ethics approval for this study was obtained from the Zhuhai People’s Hospital Institutional Review Board with approval number [2016 trial (the institute) in subclause (10)].

### Cell culture

Two human HCC cell lines (Huh7 and SK-Hep-1) were obtained from the Cell Bank of Type Culture Collection (Chinese Academy of Sciences, Shanghai, China). Cells were cultured with Dulbecco’s modified Eagle’s medium (Invitrogen, Carlsbad, CA, USA) containing 10% fetal bovine serum (FBS; Invitrogen) and incubated at 37 °C with 5% CO_2_.

### Gene- and miRNA-expression datasets

Data regarding miR-125b-5p levels from 372 HCC patients (follow-up information was included for 328 patients), and data regarding TXNRD1 levels from 351 HCC patients (concurrent data concerning miR-125b-5p levels were included for 347 patients) were obtained from The Cancer Genome Atlas (TCGA; https://tcga-data.nci.nih.gov/tcga/). Additionally, five microarray datasets (GSE22058, GSE54751, GSE21362, GSE12717, and GSE10694) were obtained from the Gene Expression Omnibus (GEO; http://www.ncbi.nlm.nih.gov/geo).

### Gene set enrichment analysis (GSEA)

GSEA was performed to identify gene sets or pathways associated with miR-125b-5p levels in the TCGA dataset. The 347 samples, for which miRNA- and mRNA-expression profiles were available, were used for GSEA after being divided into two groups: miR-125b-5p high or low expression. The gene pool, including the RAMASWAMY metastasis-associated gene set (http://software.broadinstitute.org/gsea/msigdb/cards/RAMASWAMY_METASTASIS_UP), was a gift from Zhongshan University (Guangzhou, China). We used GSEA software (v.2.0; http://www.broadinstitute.org/gsea), with *P *< 0.05 considered statistically significant.

### miRNA transfection

miR-125b-5p mimic**/**inhibitor was purchased from RiboBio (Guangzhou, China). A miR mimic**/**inhibitor was transfected into HCC cells upon their reaching 20% to 30% confluence and using Lipofectamine RNAiMAX (Invitrogen) at a working concentration of 100 nM according to manufacturer instructions. Cells were used for analysis after a 24- to 72-h culture.

### Plasmid transfection

Huh7, SK-Hep-1, and HEK293T cells were placed in a plate (Nest Biotechnology, Wuxi, China) and transfected with plasmids upon reaching 80% to 90% confluence using Lipofectamine 3000 (Invitrogen) according to manufacturer instructions, after with cells were used for analyses after 48 h. The *TXNRD1*-expression plasmid and those harboring either wild-type (WT) or mutant versions of the *TXNRD1* 3′ untranslated region (UTR) were obtained from ViGene Biosciences (Shandong, China).

### Luciferase-reporter assay

The fragment of the *TXNRD1* 3′ UTR containing the miR-125b-5p binding site (named WT) and fragment containing the site modified by site-directed mutagenesis (named Mutant) were cloned into the psiCHECK-2.0 vector (Promega, Madison, WI, USA). For the 3′ UTR dual-luciferase reporter assay, miR-125b-5p-overexpressing or -silenced HEK293 cells were co-transfected with WT or Mutant plasmids. Luciferase activity was detected using the luciferase reporter assay kit (Promega) at 72-h post-transfection.

### Quantitative reverse transcription polymerase chain reaction (qRT-PCR)

RNA was acquired using an RNA extraction kit (Invitrogen) according to manufacturer instructions, and qRT-PCR was performed using a SYBR Green PCR kit (Takara, Dalian, China), with *glyceraldehyde 3*-*phosphate dehydrogenase* and U6 used as internal controls for protein-coding genes and miR-125b-5p, individually. Primer specificity was verified prior to use, and assays were performed in triplicate. Primer sequences of primer are listed in Additional file 1: Table S1.

### Western blotting

HCC cells were lysed on ice using radioimmunoprecipitation assay buffer (Beyotime, Nanjing, China) containing phosphatase and protease inhibitors (Roche, Basel, Switzerland). Total protein was quantitated using the Bradford assay (Thermo Fisher Scientific, Waltham, MA, USA). Equivalent protein lysates were subjected to 10% sodium dodecyl sulfate polyacrylamide gel electrophoresis and then transferred onto a polyvinylidene fluoride membrane that was blocked using 5% nonfat milk for 40 min at 37 °C, followed by incubation at 4 °C for > 8 h with antibodies against TXNRD1 (Proteintech, Rosemont, IL, USA) and horseradish peroxidase-conjugated secondary antibodies (Cell Signaling Technology, Danvers, MA, USA). Peroxidase activity was visualized using an enhanced chemiluminescence substrate (Bio-Rad Laboratories, Hercules, CA, USA).

### Cell Counting Kit (CCK)-8 assay

Cells were placed in 96-well plates (~ 800 cells/well; Nest Biotechnology, Jiangsu, China), and 10 μL of CCK-8 solution (Dojindo Laboratories, Osaka, Japan) was added to each well at 24-, 48-, 72-, 96-, or 120-h post-treatment. After 2-h incubation in the dark, absorbance was detected at 450 nm.

### 5-ethynyl-2′-deoxyuridine (EdU)-incorporation assay

Cells in 96-well plates at a density of 5000 cells/well were analyzed utilizing a Cell-Light EdU Apollo 567 kit (RiboBio) according to the manufacturer’s instructions. We used an inverted fluorescence microscope (Olympus, Tokyo, Japan) to count cells in five random fields, with experiments performed in triplicate using independent samples.

### Transwell-migration and Boyden-invasion assays

For the Transwell-migration assay, ~ 1 × 10^5^ cells suspended in 100 µL of serum-free medium were placed in the upper layer of the Transwell (Corning Life Sciences, Lowell, MA, USA) while the lower layer received 500 µL of medium containing 10% FBS. We washed the inserts three times using phosphate-buffered saline, and fixed cells that adhered to the Transwell were incubated with methanol for ~ 10 min at room temperature, followed by dying with crystal violet for 20 min. Experiments were performed in triplicate. Boyden-invasion assay were performed using the same protocol as that for the cell-migration assay, but the membrane used was coated with 24 mg/mL Matrigel (BD Biosciences, Franklin Lakes, NJ, USA).

### Statistical analysis

SPSS software (v.16.0; SPSS Inc., Chicago, IL, USA) was used for data analysis. Data represent the mean ± standard error of the mean of at least three replicates. Student’s *t* test was used to evaluate differences between two groups, and one-way analysis of variance (ANOVA), followed by Dunnett’s multiple comparison test was used to assess differences between multiple groups. The results of the CCK-8 assay were evaluated using multi-way classification ANOVA, and overall and relapse-free survival rates were analyzed using the Kaplan–Meier method [12]. Correlations were analyzed with Pearson’s correlation. A *P* < 0.05 was considered statistically significant.

## Results

### miR-125b-5p levels are downregulated in HCC tissues and cell lines

**Fig. 1:**
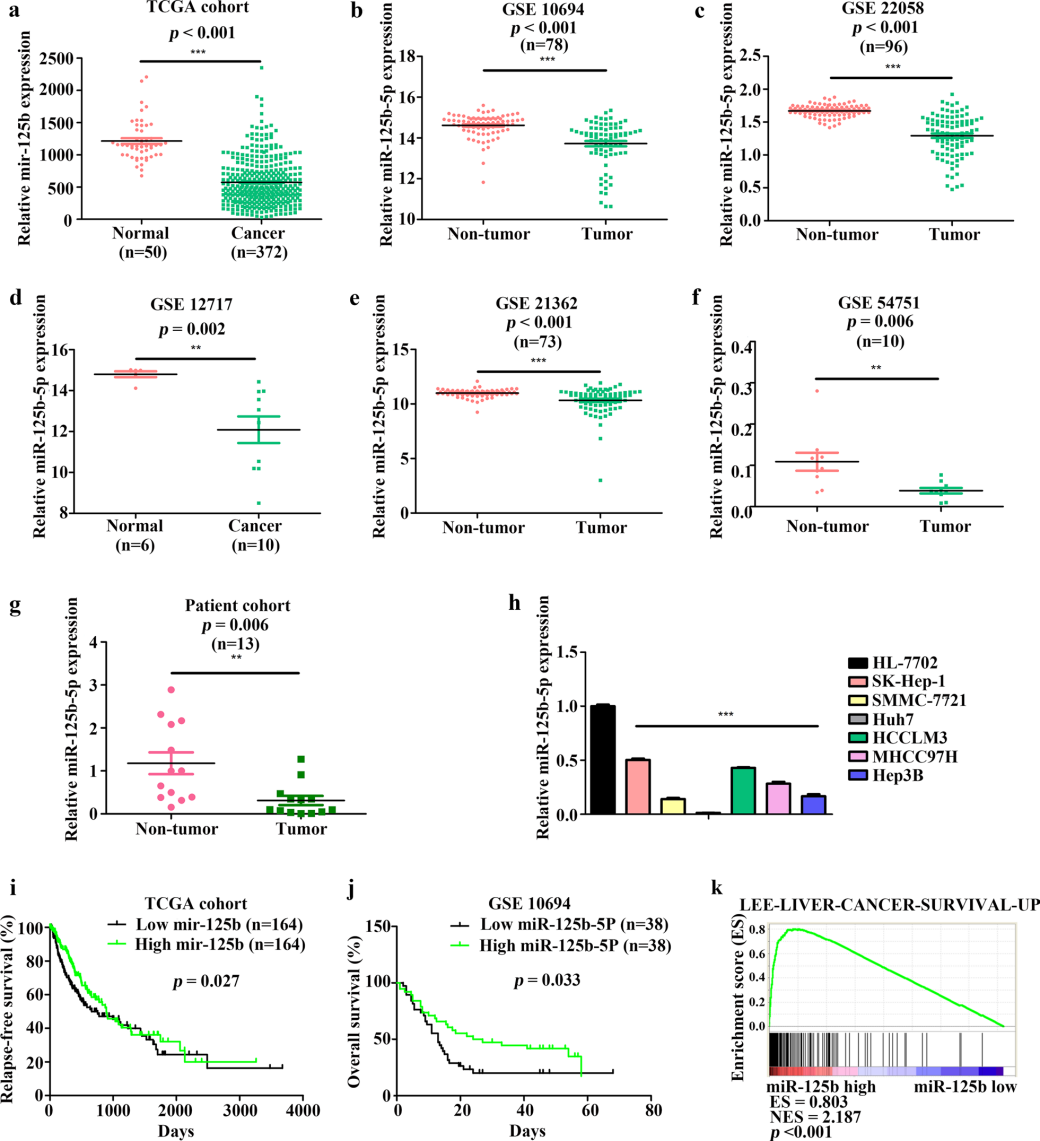
miR-125b-5p levels are attenuated in HCC tissues. miR-125b-5p levels in HCC and non-tumor tissues in the **a** The Cancer Genome Atlas (TCGA) cohort and five Gene Expression Omnibus (GEO) datasets [**b** GEO series (GSE)10694, **c** GSE22058, **d** GSE12717, **e** GSE21362, and **f** GSE54751] (*P* < 0.001, *P* < 0.001, *P* < 0.001, *P* = 0.002, *P* < 0.001, and *P* = 0.006, respectively; Student’s *t* test). miR-125b-5p levels according to qRT-PCR in **g** an HCC cohort (*n* = 13; *P* = 0.006; Student’s *t* test) and **h** normal liver cells and HCC cell lines. miR-125b-5p expression is associated with **i** relapse-free survival of HCC patients in the TCGA cohort and **j** the overall survival of HCC patients in the GSE10694 dataset (*P* = 0.027 and *P* = 0.033, respectively; Kaplan–Meier analysis). **k** HCC patients exhibiting elevated miR-125b-5p expression showed better overall survival than patients with low miR-125b-5p expression according to gene set enrichment analysis (GSEA) of the TCGA cohort (Enrichment score = 0.803, *P* < 0.001)

We found that miR-125b-5p levels were significantly reduced in HCC tissues relative to those in precancerous non-tumor or normal liver tissues, according to data from five GEO datasets and a TCGA cohort (Fig. 1a–f). Additionally, qRT-PCR analysis showed that miR-125b-5p levels were lower in HCC tissues relative to those in corresponding non-cancerous tissues (*n* = 13; *P* = 0.006) (Fig. 1g). Moreover, we confirmed that miR-125b-5p levels were lower in six HCC cell lines (SK-Hep-1, SMMC-7721, Huh7, HCCLM3, MHCC97H, and Hep3B) as compared with that in a normal liver cell line (HL-7702) (Fig. 1h). Furthermore, Kaplan–Meier survival analysis indicated that HCC patients exhibiting low miR-125b-5p expression had a poor prognosis in a TCGA cohort (*P* = 0.027) (Fig. 1i) and the GSE10694 dataset (*P* = 0.033) (Fig. 1j). GSEA showed miR-125b-5p expression was positively associated with gene signatures related to HCC-patient survival in the TCGA cohort (Fig. 1k). These results indicated that miR-125b-5p expression was downregulated in HCC samples and associated with HCC-patient prognosis.

### miR-125b-5p inhibits HCC cell proliferation, migration, and invasion

**Fig. 2:**
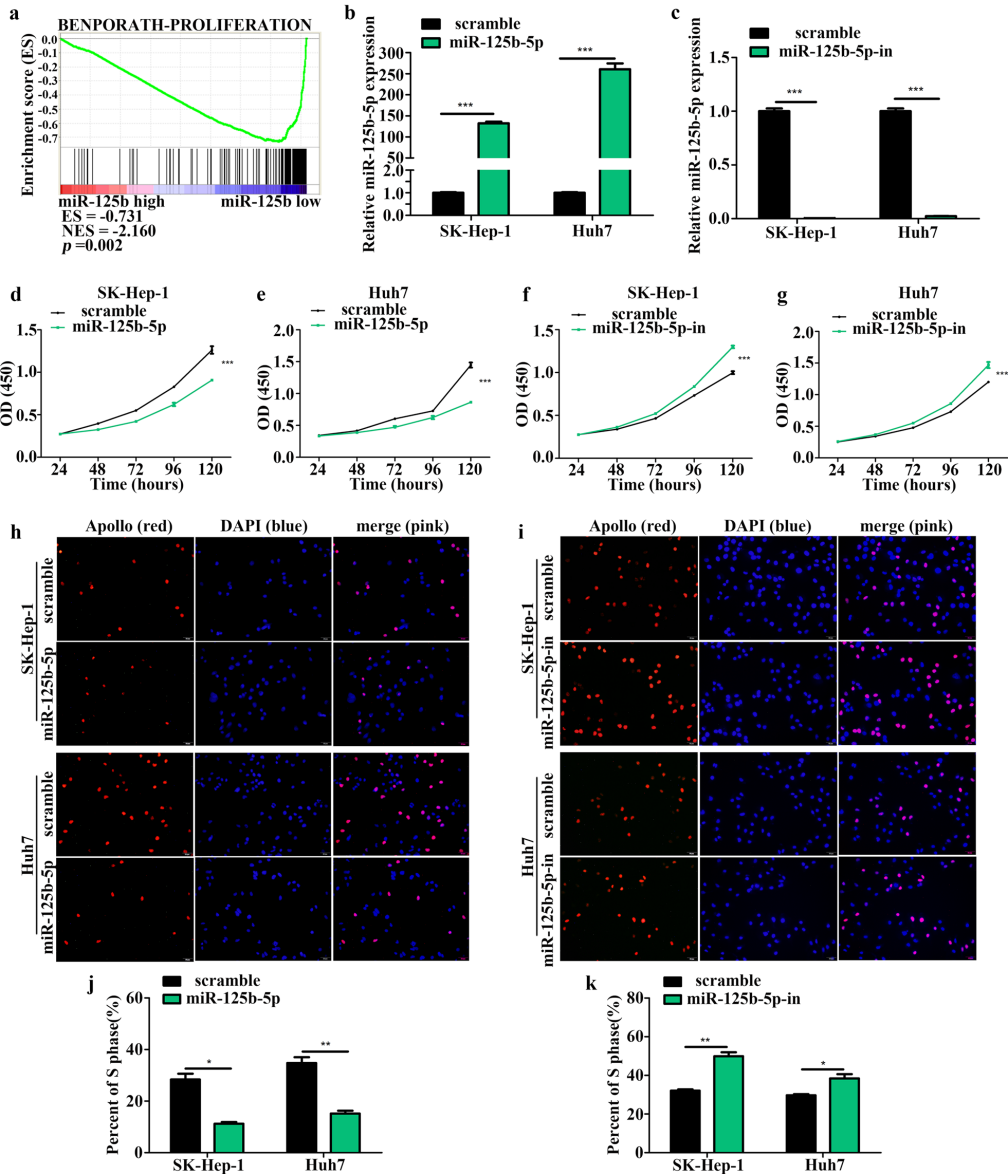
miR-125b-5p inhibits HCC cell proliferation. **a** miR-125b-5p levels were negatively associated with HCC cell proliferation according to GSEA of the TCGA cohort (enrichment score = − 0.731; *P* = 0.002). Transfection efficiency of the **b** miR-125b-5p mimic and **c** inhibitor in HCC cells according to qRT-PCR (*n *= 3; Student’s *t* test). **d**–**g** Proliferation of HCC cells transfected with miR-125b-5p mimic or inhibitor according to CCK-8 analysis (*n *= 3; factorial design ANOVA). EdU assays showing the proportion of S-phase cell after transfection of **h** miR-125b-5p mimic or **i** inhibitor. Nuclei were stained with 4′,6-diamidino-2-phenylindole, and nuclei of S-phase cells were stained with Apollo dye to react with EdU. **j**, **k** Statistical analysis of EdU incorporation (*n *= 3; Student’s *t* test). *P < 0.05; **P < 0.01; ***P < 0.001

**Fig. 3:**
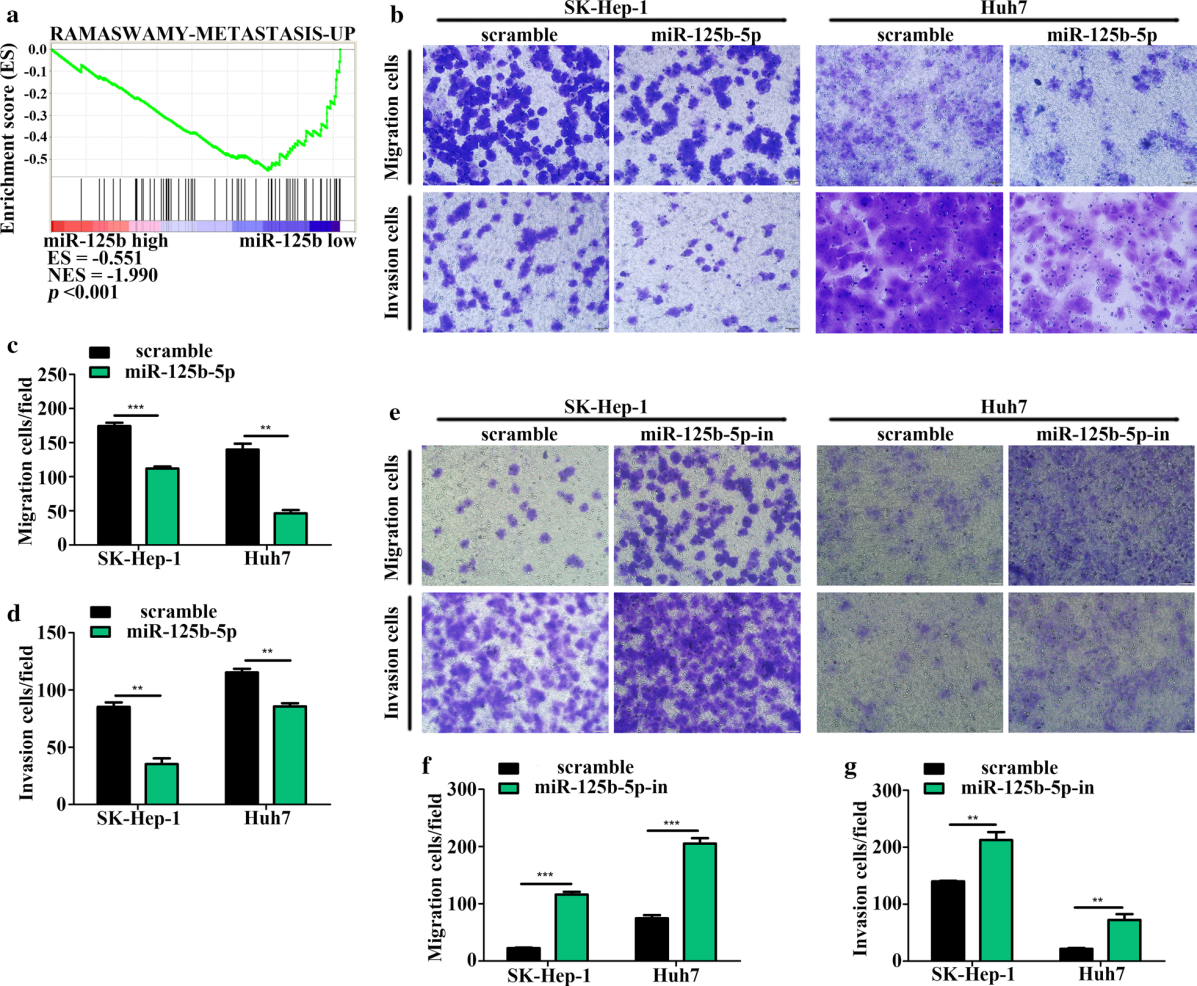
miR-125b-5p inhibits HCC cell migration and invasion. **a** miR-125b-5p expression was negatively associated with metastasis according to GSEA of the TCGA cohort (enrichment score = − 0.551; *P* < 0.001). **b** HCC cell migration and invasion were evaluated in cells transfected with the miR-125b-5p mimic and using Transwell and Boyden assays. **c**, **d** Statistical analysis of Transwell and Boyden assay results (*n *= 3; Student’s *t* test). **e** HCC cell migration and invasion were evaluated in cells transfected with miR-125b-5p inhibitor and using Transwell and Boyden assays. **f**, **g** Statistical analysis of Transwell and Boyden assay results (*n *= 3; Student’s *t* test). *P < 0.05; **P < 0.01; ***P < 0.001

To examine the biological roles of miR-125b-5p in HCC, we performed GSEA of the TCGA cohort, which indicated that miR-125b-5p might be involved in regulating HCC cell proliferation (Fig. 2a). We then transfected Huh7 and SK-Hep-1 cells with miR-125b-5p mimic/inhibitor, followed by qRT-PCR assays to verify transfection efficiency (Fig. 2b, c). We found that miR-125b-5p overexpression suppressed Huh7 and SK-Hep-1 cell proliferation according to CCK-8 assays (Fig. 2d, e), whereas miR-125b-5p silencing had the opposite effect (Fig. 2f, g). Additionally, EdU-incorporation assays suggested that exogenous introduction or knockdown of miR-125b-5p in HCC cells reduced or increased the percentage of S-phase cells, respectively (Fig. 2h–k). Moreover, GSEA of the TCGA cohort revealed miR-125b-5p levels as negatively correlated with metastasis-related gene sets associated with HCC (Fig. 3a). Furthermore, Transwell and Boyden assays showed that miR-125b-5p upregulation attenuated the migratory ability and invasiveness of Huh7 and SK-Hep-1 cells (Fig. 3b–d), whereas miR-125b-5p knockdown resulted in the opposite effects (Fig. 3e–g), suggesting that miR-125b-5p inhibited the migration and invasion of HCC cells. These data indicated that ectopic expression of miR-125b-5p inhibited the proliferation, migration, and invasion of HCC cells.

### *TXNRD1* is a direct target of miR-125b-5p

**Fig. 4:**
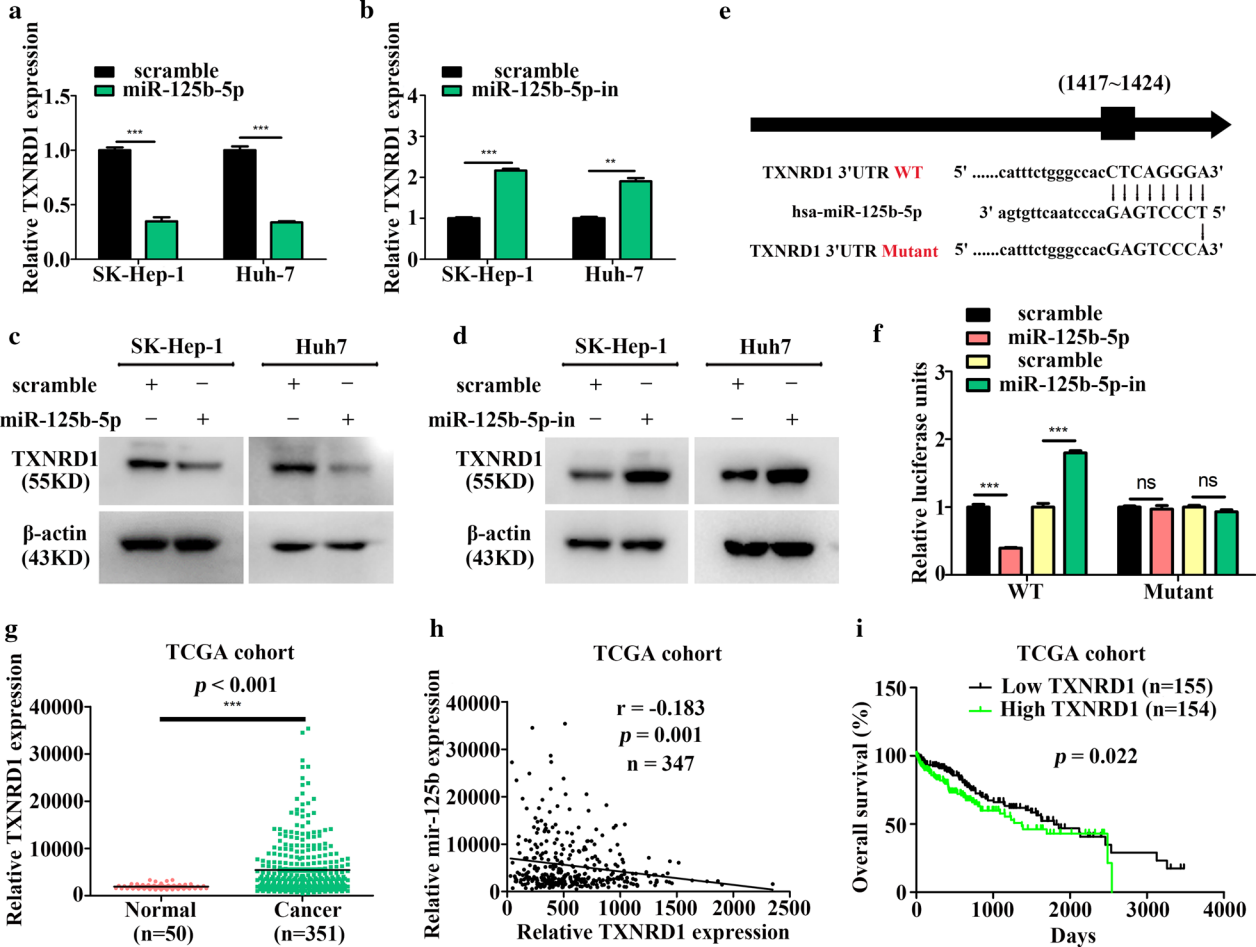
miR-125b-5p targets *TXNRD1*. **a**, **b** qRT-PCR and **c**, **d** western blot analyses of TXNRD1 levels in HCC cells transfected with the miR-125b-5p mimic or inhibitor (*n *= 3; Student’s *t* test). **e** Diagram of the putative miR-125b-5p-binding site in the *TXNRD1* 3′ UTR. **f** Luciferase-reporter assay verifying miR-125b-5p targeting of *TXNRD1* in HEK293T cells (*n *= 3; Student’s *t* test). **g** TXNRD1 levels in HCC and non-tumor samples from the TCGA cohort (*P* < 0.001; Student’s *t* test). **h** *TXNRD1* expression was inversely correlated with miR-125b levels according to data from HCC patients in the TCGA cohort (*P* = 0.001; Pearson correlation). **i** *TXNRD1* expression was associated with overall survival of HCC patients according to analysis of the TCGA cohort (*P* = 0.022; Kaplan–Meier analysis). *P < 0.05; **P < 0.01; ***P < 0.001

To determine the mechanisms associated with the inhibitory effects of miR-125b-5p on HCC cell proliferation, migration, and invasion, we used the online website TargetScan (http://www.targetscan.org/) and miRDB (http://www.mirdb.org/) to predict potential miR-125b-5p targets. Following literature review, we investigated targets reportedly upregulated in HCC according to the TGCA cohort, including *TXNRD1*, *protein phosphatase Mg*^*2*+^*/Mn*^*2*+^-*dependent*-*1F*, and *Cbl proto*-*oncogene B*. qRT-PCR analysis of their mRNA levels in miR-125b-5p-overexpressing or silenced HCC cells revealed that only *TXNRD1* was regulated by miR-125b-5p (Fig. 4a, b). Moreover, western blot analysis of the same cells showed decreased or increased TXNRD1 levels in SK-Hep-1 and Huh7 cells depending on miR-125b-5p overexpression or knockdown, respectively (Fig. 4c, d). These results suggested that TXNRD1 might be targeted by miR-125b-5p.

To verify this hypothesis, we co-transfected luciferase-reporter plasmids harboring either a WT or mutant version of the *TXNRD1* 3′ UTR with the miR-125b-5p mimic/inhibitor into HEK293T cells. We observed reduced luciferase activity in 3′ UTR WT—but not mutant-transfected cells overexpressing miR-125b-5p, whereas cells harboring silenced miR-125b-5p displayed the opposite effects, indicating that miR-125b-5p directly modulated *TXNRD1* levels by targeting its 3′ UTR (Fig. 4e, f). Furthermore, analysis of HCC cohorts for TCGA showed that *TXNRD1* level were upregulated in HCC samples and negatively correlated with miR-125b-5p expression (Fig. 4g, h). Moreover, HCC patients exhibiting elevated *TXNRD1* levels showed a poor prognosis in the TCGA cohort (*P* = 0.022) (Fig. 4i). These data suggested *TXNRD1* as a direct target of miR-125b-5p.

### miR-125b-5p modulates HCC cell proliferation, migration, and invasion by targeting *TXNRD1*

**Fig. 5:**
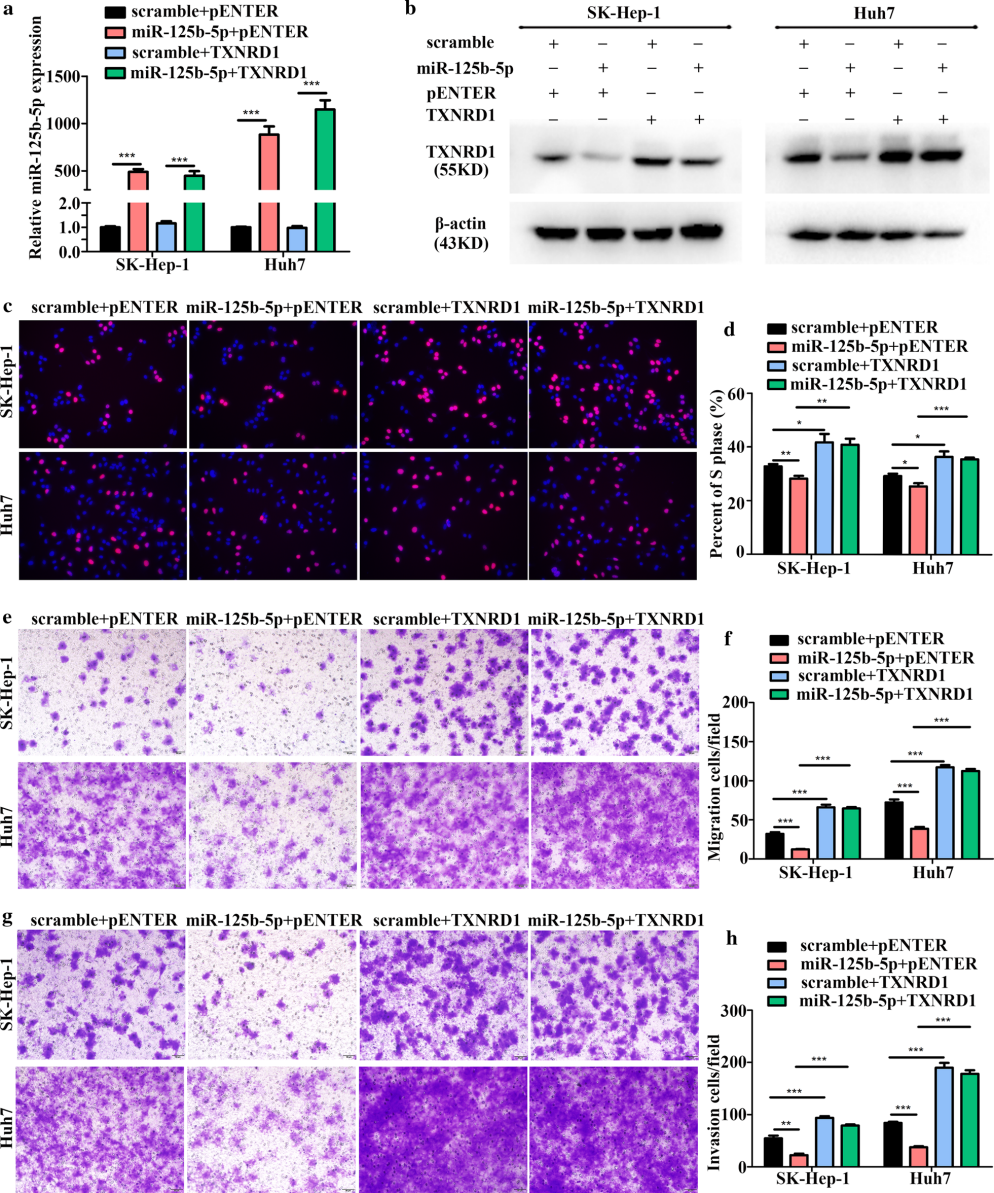
*TXNRD1* overexpression counteracts miR-125b-5p-mediated attenuation of HCC cell migration and invasion. **a** qRT-PCR- and **b** western blotting-based confirmation of miR-125b-5p and *TXNRD1* overexpression. pENTER (empty plasmid) was used as a control to determine *TXNRD1* overexpression (*n *= 3; one-way ANOVA and Dunnett’s multiple comparison test). **c**–**h** *TXNRD1* overexpression abolished miR-125b-5p-mediated repression of **c**, **d** HCC cell proliferation according to EdU-incorporation assay (*n *= 3; one-way ANOVA and Dunnett’s multiple comparison test) and **e**–**h** migration and invasion in HCC cells (*n *= 3; one-way ANOVA and Dunnett’s multiple comparison test). *P < 0.05; **P < 0.01; ***P < 0.001

**Fig. 6:**
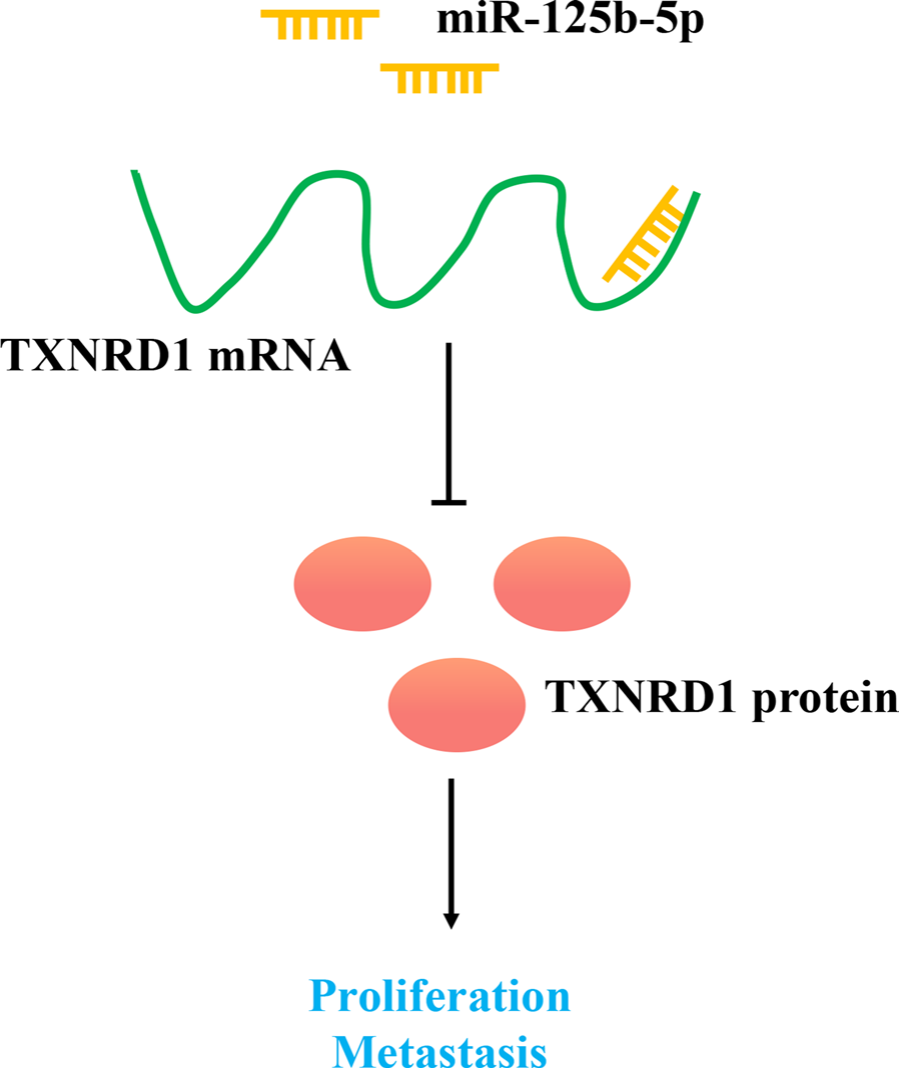
Schematic of the proposed mechanism of miR-125b-5p-mediated HCC suppression. miR-125b-5p downregulates TXNRD1 level by directly targeting *TXNRD1* mRNA to inhibit HCC cell proliferation, migration, and invasion

Because *TXNRD1* is directly targeted by miR-125b-5p, we presumed that TXNRD1 also mediates miR-125b-5p function. To test this hypothesis, we overexpressed *TXNRD1* in HCC cells transfected with the miR-125b-5p mimic (Fig. 5a, b), and found that *TXNRD1* overexpression reversed miR-125b-5p-mediated suppression of HCC cell proliferation, migration, and invasion according to EdU-incorporation, Transwell, and Boyden assays, respectively (Fig. 5c–h). These results indicated that miR-125b-5p modulated the proliferation, migration, and invasion of HCC cells by targeting *TXNRD1* (Fig. 6).

## Discussion

In this study, we found that miR-125b-5p expression was attenuated in HCC tissues according to the analysis of five GEO datasets and a TCGA cohort, suggesting its possible involvement in regulating HCC progression. Although one study indicated its role in tumor progression [13], the overwhelming majority of reports suggest that miR-125b-5p as a tumor suppressor inhibits multiple biological functions in a variety of tumors, including those associated with HCC [14–19]. Additionally, previous studies report that miR-125b-5p suppresses HCC proliferation and metastasis by regulating the expression of downstream signaling molecules [17–22]. The findings of present study confirmed, for the first time, that miR-125b-5p targets *TXNRD1* to inhibit HCC cell proliferation, migration, and invasion by downregulating TXNRD1 expression.

To identify potential miR-125b-5p targets in HCC cells, we screened predicted targets forecasted as upregulated in HCC tissues according to a TCGA cohort and reported as cancer-promoting genes in HCC in the literature. qRT-PCR analysis and co-transfection of HCC cells with a miR-125b-5p mimic or inhibitor revealed TXNRD1 as the target. Previous studies identified TXNRD1 as a regulator of endocellular oxidative stress and a promoter of tumor development [23, 24], with irreversible suppression of cytoplasmic TXNRD1 levels demonstrated as a novel direction for cancer treatment [11, 25, 26]. Additionally, studies reported that TXNRD1 levels are upregulated in HCC and negatively correlated with HCC-patient prognosis [27, 28]. In the present study, our data demonstrated that TXNRD1 promoted the proliferation, migration, and invasion of HCC cells, which provided insight into the previously unknown molecular mechanisms associated with TXNRD1 in HCC. Metabolic analysis showed that TXNRD1 is crucial for the final step in the synthesis of nucleic acids by providing reductive equivalents to ribonucleotide reductase [29], and failure to maintain sufficient stores of 2′-deoxyribonucleotides resulted in damaged DNA and cell cycle arrest in *TXNRD1*-deficient T cells [30]. These findings and our results from the present study suggest that the mechanisms underlying TXNRD1 regulation of HCC status might be associated with oxidative stress or nucleotide biosynthesis; however, further studies are required to clarify these roles.

Here, we demonstrated the tumor-suppressive property of miR-125b-5p via its targeting of *TXNRD1*, which broadens our understanding of the molecular mechanism associated with miR-125b-5p-related functions in HCC cells. Nevertheless, the mechanism underlying the attenuated expression of miR-125b-5p in HCC cells remains to be investigated in future research.

## Conclusions

This represents the first study reporting that *TXNRD1* is directly targeted by miR-125b-5p. Our results indicated that miR-125b-5p represents a tumor suppressor in HCC through its attenuation of TXNRD1 levels, thereby suggesting this miRNA as a potential target for HCC treatment.

## Additional file

**Additional file 1: Table S1.** Primer sequences used in this study.

## Data Availability

The datasets used and/or analyzed in this study are available from the corresponding author upon reasonable request.

## Abbreviations

CCK-8: cell counting kit-8
EDU: 5-ethynyl-2′-deoxyuridine
FBS: fetal bovine serum
GEO: gene expression omnibus
GSEA: gene set enrichment analysis
HCC: hepatocellular carcinoma
miRNAs: microRNAs
qRT-PCR: quantitative reverse transcription polymerase chain reaction
TCGA: The Cancer Genome Atlas
TRX1: thioredoxin 1
TXNRD1: thioredoxin reductase 1
UTR: untranslated region
