# Supplementary material for: miR-125b-5p inhibits cell proliferation, migration, and invasion in hepatocellular carcinoma via targeting TXNRD1
Source: Cancer Cell Int. 2019 Jul 30;19:203. doi: 10.1186/s12935-019-0919-6 (PMC6668076; doi:10.1186/s12935-019-0919-6)
Supplement: Supplementary file 1 — Additional file 1: Table S1. Primer sequences used in this study. [file 12935_2019_919_MOESM1_ESM.docx]

**Additional Table S1**

**Primer sequences used in this study**

| **Type** | **Gene** | **Sequence** |
| --- | --- | --- |
| **Primers** | **miR-125b-5p**  **U6**  **TXNRD1**  **PPM1F**  **CBLB** | **F 5’** **ACACTCCAGCTGGGTCCCTGAGACCCTAAC 3’**  **R 5’CTCAACTGGTGTCGTGGAGTCGGCAATTCAGTTGAGTCACAAGT3’**  **F 5’CTCGCTTCGGCAGCACA3’**  **R 5’ AACGCTTCACGAATTTGCGT 3’**  **F 5’** **GTTGCAATCCAGGCAGGAAG3’**  **R 5’** **CCCACAACACGTTCATTGTCTT3’**  **F 5’** **CGGAGACCAGGGGGTGAA3’**  **R 5’** **ATTGGGCTGCTCTTCTGTGG3’**  **F 5’** **CCATCATGTGGAAAGCGTGC3’**  **R 5’** **GTGACCATTGGAAAAGACCTTAGC3’** |
